# CORRECTING GLUTATHIONE DEFICIENCY AND MITOCHONDRIAL DYSFUNCTION IN OLDER HUMANS: A RANDOMIZED CLINICAL TRIAL

**DOI:** 10.1093/geroni/igz038.1552

**Published:** 2019-11-08

**Authors:** Rajagopal V Sekhar, Premranjan Kumar, Jean W Hsu, James Suliburk, George E Taffet, Charles G Minard, Farook Jahoor, Chun Liu

**Affiliations:** Baylor College of Medicine, Houston, Texas, United States

## Abstract

Aging is associated with impaired mitochondrial fatty-acid oxidation (MFO) due to unknown mechanisms, and interventions are lacking. We hypothesized that impaired MFO in aging occurs due to Glutathione-deficiency and tested this in a randomized, placebo-controlled double-blind clinical-trial in 24 older-humans (71.1y) and 12 young-controls (25.5y) using calorimetry, muscle-biopsy and tracer-protocols. Older-humans received either GlyNAC (Glycine 1.33mmol/kg/d and N-acetylcysteine 0.83mmol/kg/d as Glutathione precursors) or isonitrogenous-placebo for 16-weeks; young-controls received GlyNAC for 2-weeks. Compared to young-controls, older humans had significantly lower Glutathione, impaired MFO, lower gait-speed and physical-function, and higher oxidative-stress, inflammation and insulin-resistance. GlyNAC supplementation in older-humans significantly improved and restored MFO; increased gait-speed (19%,) and physical-function; and decreased oxidative-stress (TBARS 80%), inflammation (IL-6 83%; TNF-alpha 58%), and insulin-resistance (HOMA-IR 68%), but young-controls were unaffected. These data provide proof-of-concept that GlyNAC supplementation could improve the health of older-humans by correcting Glutathione-deficiency and mitochondrial-defects to improve gait-speed, oxidative-stress, inflammation and insulin-resistance.

